# Atopic Dermatitis and Markers of Early Cardiovascular Risk in Children and Adolescents

**DOI:** 10.1001/jamanetworkopen.2026.2962

**Published:** 2026-03-24

**Authors:** Morgan Ye, Charles E. McCulloch, Carlos Iribarren, Sinéad M. Langan, Katrina Abuabara

**Affiliations:** 1 Department of Dermatology, University of California, San Francisco; 2 Department of Epidemiology and Biostatistics, University of California, San Francisco; 3 Department of Research, Kaiser Permanente, Oakland, California; 4 Faculty of Epidemiology and Population Health, London School of Hygiene and Tropical Medicine, London, United Kingdom

## Abstract

**Importance:**

Early identification of individuals at elevated risk of cardiovascular disease and preventive treatment during childhood may reduce cardiovascular disease in adults with chronic inflammatory diseases. Children with atopic dermatitis (AD) may have elevated cardiovascular risk, but studies to date have not accounted for heterogeneity in disease activity and severity across childhood.

**Objectives:**

To evaluate whether active and more severe AD across childhood and adolescence are associated with cardiovascular risk.

**Design, Setting, and Participants:**

This longitudinal cohort study used data collected from 1991 to 2017 for the Avon Longitudinal Study of Parents and Children, a population-based birth cohort in the United Kingdom. Participants included children alive at 1 year with assessment of AD and at least 1 cardiovascular disease risk factor at a minimum of 1 time point. Data were analyzed November 30, 2022, to February 20, 2025.

**Exposures:**

Repeated assessments of AD activity and severity across childhood and adolescence.

**Main Outcomes and Measures:**

The primary outcome was cardiometabolic risk scores calculated at ages 15, 17, and 24 years. Secondary outcomes included body mass index, blood pressure, and lipid profiles measured up to 12 times between ages 3 and 24 years and ultrasonography measures of subclinical atherosclerosis at ages 17 and 24 years.

**Results:**

The subcohort included 9281 children, of whom 4669 (50.31%) were male. The analysis included 1001 participants (10.79%) 3 years of age, 908 (9.78%) 4 years, 838 (9.03%) 5 years, 6352 (68.44%) 7 years, 6205 (66.86%) 10 years, 5629 (60.65%) 11 years, 4968 (53.53%) 13 years, 3502 (37.73%) 15 years, 4738 (51.05%) 17 years, and 3626 (39.07%) 24 years of age. The prevalence of active AD varied by age and ranged from 13.10% to 21.58% at ages 3 through 18 years. Among participants with AD, 3.52% to 6.85% reported moderate or severe disease at each age. Multivariable linear regression models did not reveal associations between active AD and most cardiovascular risk factors; only 2 associations between AD and low-density lipoprotein cholesterol levels were found with *P* < .05, but they differed in the directionality of association at ages 3 and 10 years (mean difference, −0.33 [95% CI, −0.58 to −0.07] SDs for 3 years vs 0.14 [95% CI, 0.03-0.24] SDs at 10 years). There was no consistent evidence for dose-response effects by AD severity. There were also no associations between patterns of more active and severe AD across childhood with subclinical atherosclerosis.

**Conclusions and Relevance:**

In a population-based UK cohort of children and adolescents followed up into early adulthood, AD, including more active and severe disease over time, was not associated with increases in markers of cardiovascular risk. This finding suggests that systematic screening of all children with AD is unlikely to improve identification of early cardiovascular risk.

## Key Points

**Question:**

Is more active and severe atopic dermatitis (AD) throughout childhood associated with early cardiovascular risk factors?

**Findings:**

In this cohort study including 9281 participants, there were no associations between AD and most cardiovascular risk factors, no consistent evidence for a dose response by AD severity, and no associations between trajectories of more active and severe AD with measures of subclinical atherosclerosis and cardiometabolic risk.

**Meaning:**

Findings indicating that children with AD did not have early signs of increased cardiovascular risk suggest that standardized screening of all children with AD is unlikely to improve identification of those in need of early intervention.

## Teaser

This cohort study investigates whether active and more severe atopic dermatitis across childhood and adolescence are associated with cardiovascular risk.

## Introduction

An increasing body of evidence supports an association between atopic dermatitis (AD, also known as atopic eczema) and cardiovascular disease (CVD). Studies of adults have found that AD, and in particular severe AD, is associated with a higher risk of myocardial infarction, stroke, and heart failure.^1,2^ Other studies have found associations between AD and image-based and blood-based markers of cardiovascular risk.^3,4^ However, some studies have found no association, and no guidelines or recommendations for screening exist to date.^5^

Cardiovascular risk factors, including hypertension, dyslipidemia, and obesity in childhood and adolescence, have been shown to be highly predictive of later life cardiovascular morbidity and mortality. For example, the international i3C consortium followed up 43 324 participants ages 3 to 19 years of age into adulthood and found that an SD increase in the combined *z* score for 5 risk factors (body mass index [BMI], systolic blood pressure, total cholesterol level, triglyceride level, and youth smoking) in childhood increased the risk of adult cardiovascular events by nearly 3-fold.^6^ Additionally, subclinical evidence of atherosclerotic disease based on noninvasive imaging and measures of endothelial function can be detected in adolescence and early adulthood and are important predictors of long-term risk of vascular disease.^7^

Limited data suggest that children with AD may have higher rates of cardiovascular risk factors, but most studies have been cross-sectional in nature and have not adequately adjusted for factors such as BMI, which is an established risk factor for CVD. Multiple observational and mendelian randomization studies have found associations between BMI or measures of obesity and AD; therefore, it is likely to be an important confounder to adjust for in analyses of AD and cardiovascular risk.^8,9^ Additional research is needed to identify children at highest risk because targeted screening and early intervention may mitigate cardiovascular risk and mortality. The objectives of this study were to evaluate whether AD is associated with cardiovascular risk factors in children and to assess whether more active and severe AD across childhood is associated with cardiometabolic risk and subclinical atherosclerosis in early adulthood.

## Methods

### Study Population

We used data from the Avon Longitudinal Study of Parents and Children (ALSPAC), a population-based birth cohort from the UK that recruited pregnant women with expected delivery dates of April 1, 1991, to December 31, 1992. ALSPAC initially enrolled 14 541 pregnant women, which resulted in 13 988 live infants at 1 year of age from 14 062 live births. We excluded participants who did not have any available data on AD or CVD risk factors and analyzed data from a subsample of the cohort consisting of 9281 participants with assessment of AD and at least 1 CVD risk factor at a minimum of 1 time point. There were not large differences in participant characteristics between the included and excluded participants (eTable 1 in Supplement 1). Study and data details are available on the study website.^10^ Race and ethnicity data, reported as White and non-White based on the distribution of groups and small number of participants who belonged to minoritized racial and ethnic groups, were collected as a covariate because existing literature suggests that race and ethnicity are a potential confounder in the relationship between AD and CVD risk. Informed consent was obtained from participants following the recommendations of the ALSPAC Ethics and Law Committee. Ethical approval for the study was obtained from the ALSPAC Ethics and Law Committee and the local research ethics committees. The study was exempt from institutional review board approval by the University of California, San Francisco, because investigators did not have access to identifiable data. We followed the Strengthening the Reporting of Observational Studies in Epidemiology (STROBE) reporting guideline.

### Exposure

The primary exposure was active AD, which was defined by at least 2 positive responses to the question (including at the current assessment age), “Has your child had itchy, dry skin rash in the joints and creases of the body (eg, behind the knees, elbows, or under the arms) in the past year?” This question is based on the standardized questionnaire from the International Study of Asthma and Allergies. Parents were asked about their child’s symptoms of AD at 11 time points (6, 18, 30, 42, 57, 69, 81, 103, 128, 140, and 166 months), and participants responded about their own AD symptoms at 16 and 18 years. AD severity was based on a repeated question asking participants to rank their or their child’s AD as “no problem, mild, very bad, or quite bad” at the same time points as AD symptoms. Parent- and self-reported AD severity has been shown to be similar to physician-assessed disease severity.^11^ Based on the distribution of responses, severity was grouped into clinically appropriate categories entitled no problem or mild and very bad or quite bad to ensure adequate data for severity categories.

In addition to the period prevalence of active AD and AD severity modeled at 13 time points, cumulative risk of disease activity and severity between birth and age 14 years was modeled using 5 disease trajectory phenotypes identified previously from a latent class analysis of AD in the ALSPAC database, in which individuals’ posterior class probabilities were estimated and their most likely phenotype was identified as the class with the highest probability.^12^ A total of 4% were classified into a severe-frequent AD group, 8% into a moderate-frequent AD group, 12% into a moderate-declining AD group, 10% into a mild-intermittent AD group, and 66% into an unaffected or rarely affected group.^12^

### Outcomes

Markers of cardiovascular risk were measured at 12 assessment ages between 3 and 24 years of age (eFigure 1 in Supplement 1). Blood pressure was assessed using the mean of 2 readings of systolic and diastolic blood pressure. Weight and height were measured to assess BMI. Plasma lipid concentrations, including total cholesterol, triglycerides, and high-density lipoprotein cholesterol (HDL-C), were measured using the standard Lipid Research Clinics Protocol with enzymatic reagents for lipid determination. Low-density lipoprotein cholesterol (LDL-C) concentration was determined from these measures with the Friedwald equation. The distribution and patterns of missing data were evaluated for all variables. The triglycerides measure was log-transformed to address skewness.^13^

The primary outcome was a cardiometabolic risk score that has previously been validated in the ALSPAC cohort.^14^ Cardiometabolic risk was calculated at ages 15, 17, and 24 years using the following variables: systolic and diastolic blood pressure, abdominal fat mass, fasting plasma glucose, HDL-C, and triglycerides. These variables were standardized and summed as follows: mean arterial pressure ([(2 × diastolic blood pressure) + systolic blood pressure]/3); abdominal fat mass; fasting plasma glucose; HDL-C × −1; and triglyceride.

Cardiometabolic risk score = ([(2 × diastolic blood pressure) + systolic blood pressure]/3) + abdominal fat mass + fasting plasma glucose + (−1[HDL-C]) + triglycerides

Two measures of subclinical CVD at 17 and 24 years of age were evaluated: intima media thickness and pulse wave velocity. Carotid artery intima media thickness was assessed by ultrasonography. Measurements were repeated bilaterally 1 to 2 cm proximal to the carotid bifurcation for 3 different cardiac cycles and averaged. Pulse wave velocity was measured by pressure pulse waveforms recorded via high fidelity micromanometer from the carotid pulse synchronous with an electrocardiogram signal. The mean time difference between the R-wave and the pressure wave on a beat-to-beat basis over 10 seconds was recorded, and the pulse wave was determined by the mean time difference and arterial path length between the 2 recording points (carotid to radial and carotid to femoral). All outcomes, including individual markers of cardiovascular risk and measures of subclinical CVD, were converted to *z* scores to enable comparisons between outcomes and to facilitate interpretability.

### Covariates

We identified potential confounders from the existing literature. Potential confounders include characteristics assessed at birth (biological sex at birth, birth weight, maternal age at birth, gestational age at birth, parity, and racial and ethnic group), socioeconomic status (social class based on the highest occupation of the parents recorded in an antenatal questionnaire and at the 3-year survey), maternal educational attainment, and time-updated history of tobacco smoke exposure, self-reported heavy traffic near home, other atopic diseases (history of wheeze or asthma and hay fever), and BMI.

### Statistical Analysis

We used linear regression models to examine associations between active AD and cardiometabolic risk scores at ages 15 and 17 years, adjusting for the aforementioned covariates. We also examined active AD and AD severity with each of the individual cardiovascular risk factors measured concurrently (or within 1 year) at ages 3, 4, 5, 7, 10, 11, 13, 15, and 17 years in separate models for each outcome. We then used linear regression models to examine the associations between previously identified AD disease trajectory phenotypes^12^ and each of the cardiovascular risk factors in addition to the 2 subclinical CVD measures at ages 17 and 24. We did not perform multiple testing correction and used a 2-sided nominal *P* value of .05. All analyses were conducted from November 30, 2022, to February 20, 2025, using Stata, version 18.0 (StataCorp LLC).

## Results

**Figure 1.**
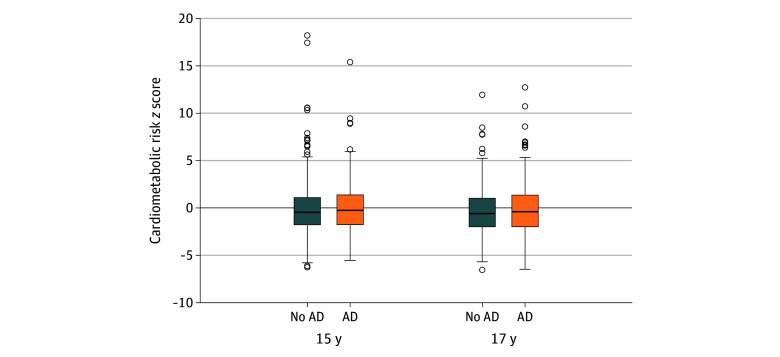
Box and Whisker Plots of Cardiometabolic Risk *z* Scores at Ages 15 and 17 Years The edges of the box represent the 25th (first quartile) and 75th (third quartile) percentiles; horizontal line through the box, the 50th percentile (median); and circles, outlier data points greater than 1.5 times the IQR above the third quartile or smaller than 1.5 times the IQR below the first quartile. AD indicates atopic dermatitis.

The sample included 9281 individuals, of whom 4612 (49.69%) were female (4669 [50.31%] male) and 8366 (95.74%) identified as White (372 [4.26%] as other racial or ethnic group) (Table). The analysis included 1001 (10.79%) participants 3 years of age, 908 (9.78%) 4 years, 838 (9.03%) 5 years, 6352 (68.44%) 7 years, 6205 (66.86%) 10 years, 5629 (60.65%) 11 years, 4968 (53.53%) 13 years, 3502 (37.73%) 15 years, 4738 (51.05%) 17 years, and 3626 (39.07%) 24 years of age. Active AD was present in 13.10% to 21.58% of participants at ages 3 to 18 years, and 3.52% to 6.85% reported moderate or severe disease at each age (eFigure 2 in Supplement 1). The mean values for cardiovascular risk factors increased with age (eFigure 3 in Supplement 1), and the distribution of values for the cardiometabolic risk score were similar between participants with vs without AD (eg, median [IQR] z score for 15 years of age, −0.47 [−1.77 to 1.10] for no AD vs −0.25 [−1.76 to 1.37] for AD) (Figure 1).

**Table.** Participant Characteristics

Characteristic	Participants, No. (%)			*P* value^a^
	Total (N = 9281)^b^	AD definition^c^		
		Did not meet (n = 5525)	Met (n = 3588)	
Sex				
Female	4612 (49.69)	2549 (46.14)	1968 (54.85)	<.001
Male	4669 (50.31)	2976 (53.86)	1620 (45.15)	
Race and ethnicity				
White	8366 (95.74)	4930 (95.71)	3311 (95.94)	.60
Other racial or ethnic group	372 (4.26)	221 (4.29)	140 (4.06)	
Low birth weight (<2500 g)				
No	8727 (95.22)	5185 (95.03)	3385 (95.43)	.39
Yes	438 (4.78)	271 (4.97)	162 (4.57)	
Maternal age at birth, y				
≤20	378 (4.07)	248 (4.49)	115 (3.21)	.01
21-34	7835 (84.42)	4655 (84.25)	3034 (84.56)	
>34	1068 (11.51)	622 (11.26)	439 (12.24)	
Preterm birth (<37 wk)				
No	8745 (94.22)	5193 (93.99)	3396 (94.65)	.19
Yes	536 (5.78)	332 (6.01)	192 (5.35)	
Parity				
0	4032 (45.66)	2389 (45.70)	1589 (46.06)	.004
1	3140 (35.56)	1807 (34.57)	1272 (36.87)	
≥2	1658 (18.78)	1031 (19.72)	589 (17.07)	
Highest parental social class at 32 weeks’ gestation + 3 y				
Professional	1539 (17.43)	841 (16.11)	685 (19.72)	<.001
Managerial and technical	4386 (49.68)	2535 (48.56)	1790 (51.54)	
Skilled nonmanual	1832 (20.75)	1146 (21.95)	656 (18.89)	
Skilled manual	805 (9.12)	513 (9.83)	265 (7.63)	
Partly skilled	223 (2.53)	156 (2.99)	63 (1.81)	
Unskilled	43 (0.49)	29 (0.56)	14 (0.40)	
Maternal educational level at 32 weeks’ gestation^d^				
CSE or none	1347 (15.14)	857 (16.31)	456 (13.04)	<.001
Vocational	825 (9.27)	497 (9.46)	310 (8.86)	
O Level	3156 (35.48)	1891 (35.98)	1207 (34.52)	
A Level	2237 (25.15)	1293 (24.60)	917 (26.22)	
Degree (≥ university)	1330 (14.95)	718 (13.66)	607 (17.36)	
Passive smoke exposure per week at 6 mo^e^				
None	6401 (74.73)	3631 (72.90)	2672 (77.97)	<.001
Low (<1 h)	922 (10.76)	546 (10.96)	353 (10.30)	
Moderate (1-2 h)	517 (6.04)	326 (6.54)	176 (5.14)	
High (≥3 h)	725 (8.46)	478 (9.60)	226 (6.59)	
Rating of traffic level near home at 8 mo^e^				
Very busy	634 (7.46)	391 (7.86)	231 (6.80)	.21
Busy	1081 (12.72)	635 (12.77)	422 (12.42)	
Moderate	2310 (27.17)	1341 (26.97)	929 (27.34)	
Quiet	2673 (31.44)	1579 (31.76)	1062 (31.25)	
Very quiet	1803 (21.21)	1026 (20.64)	754 (22.19)	
Wheeze or asthma ever up until age 18 y^e^				
No	4412 (47.55)	2900 (52.51)	1412 (39.35)	<.001
Yes	4867 (52.45)	2623 (47.49)	2176 (60.65)	
Hay fever ever up until age 18 y^e^				
No	5042 (56.84)	3398 (63.98)	1619 (45.84)	<.001
Yes	3829 (43.16)	1913 (36.02)	1913 (54.16)	

Abbreviations: AD, atopic dermatitis; CSE, Certificate of Secondary Education.

^a^ *P* values are from χ^2^ tests.

^b^ Values for each characteristic may not add up to the total because of missing data; percentages are of those with data.

^c^ Participants who did not meet the definition of AD from age 3 to 18 (ie, had at least 2 reports of flexural dermatitis), including those who never had AD or had only 1 report of flexural dermatitis. The values do not add up to the total because the total also includes individuals with data on AD disease trajectory phenotypes.

^d^ UK education levels: CSE, certificate after passing national school examinations at 16 years of age; vocational; O level, qualification after passing national school examinations at 16 years of age; A level, qualification after passing national school examinations at 18 years of age; degree, university degree, or higher.

^e^ In analytic models, this was a time-varying covariate.

**Figure 2.**
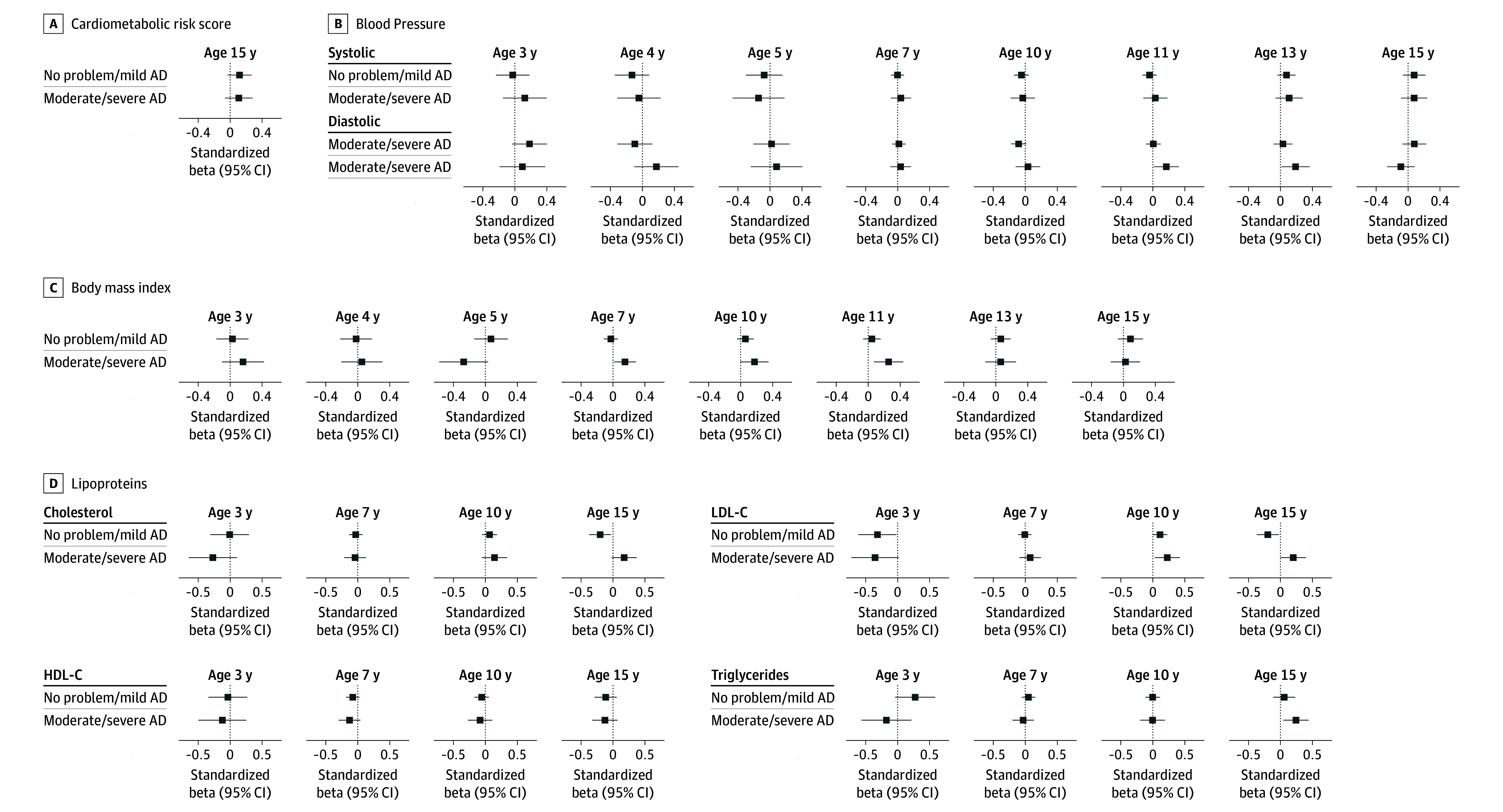
Dot Plots Showing Results of Cross-Sectional Adjusted Analyses Between Atopic Dermatitis (AD) Severity and Cardiovascular Risk Factors Linear regressions showing standardized betas in cardiovascular risk factors for no problem or mild AD and moderate or severe AD as compared with no AD (reference group). All models were adjusted for sex, race and ethnicity, birth weight, maternal age at birth, gestational age, parity, socioeconomic status, maternal educational level, ever wheeze up to age of interest, ever hay fever up to age of interest, childhood tobacco smoke exposure, and heaviness of traffic near home. All models, except for body mass index, were also adjusted for body mass index. HDL-C indicates high-density lipoprotein cholesterol; LDL-C, low-density lipoprotein cholesterol.

In unadjusted and adjusted linear regression models, we found no evidence for an association between AD and the cardiometabolic risk score at age 15 years (adjusted mean difference, 0.11 [95% CI, −0.01 to 0.24] SDs) or 17 (adjusted mean difference, 0.09 [95% CI, −0.05 to 0.23] SDs) years, including when we stratified by AD severity (Figure 2; eFigures 4 and 5, eTables 3 and 4 in Supplement 1). Similarly, we found no evidence of consistent associations between AD and the individual cardiovascular risk factors (Figure 2; eFigures 4 and 5, eTables 3 and 4 in Supplement 1). Of 49 comparisons with varying sample sizes (eTable 6 in Supplement 1), the only associations with a nominal *P* < .05 differed in directionality at 2 ages: AD was associated with increased LDL-C levels at age 10 years (mean difference, 0.14 [95% CI, 0.03-0.24] SDs) but with decreased LDL-C levels at age 3 years (mean difference, −0.33 [95% CI, −0.58 to −0.07] SDs).

### Associations Between AD Activity and Severity Subtype and CVD Risk Factors at 17 and 24 Years of Age

**Figure 3.**
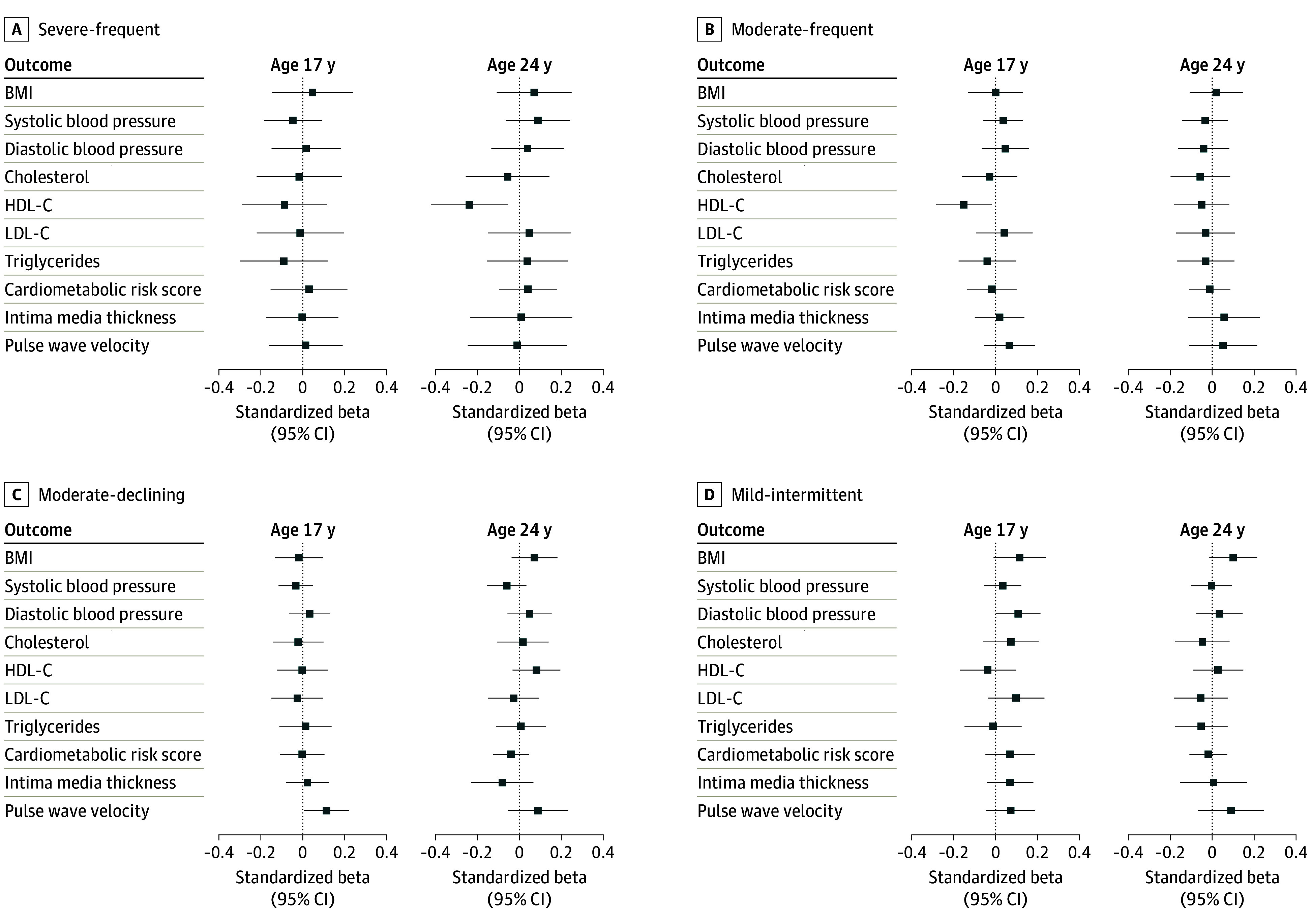
Dot Plots Showing Results of Adjusted Longitudinal Analyses Linear regressions showing standardized betas in cardiovascular risk factors for severe-frequent, moderate-frequent, moderate-declining, and mild-intermittent atopic dermatitis (AD) activity and severity groups, as compared with the unaffected-rare AD reference group. All models were adjusted for sex, race and ethnicity, birth weight, maternal age at birth, gestational age, parity, socioeconomic status, maternal educational level, ever wheeze up to age of interest, ever hay fever up to age of interest, childhood tobacco smoke exposure, and heaviness of traffic near home. All models, except for body mass index (BMI), were also adjusted for BMI. A separate model was run for each cardiovascular risk factor outcome. HDL-C indicates high-density lipoprotein cholesterol; LDL-C, low-density lipoprotein cholesterol.

We found no consistent evidence for an association between patterns of more active and severe AD with cardiovascular risk factors at ages 17 and 24 years (Figure 3; eFigure 6 and eTable 5 in Supplement 1). At age 17 years, moderate-frequent AD was associated with decreased HDL-C levels (mean difference, −0.15 [95% CI, −0.28 to −0.02] SDs), and at age 24 years, severe-frequent AD was associated with decreased HDL-C levels (mean difference, −0.24 [95% CI, −0.42 to −0.05] SDs). At age 17 years, mild-intermittent AD was associated with increased diastolic blood pressure (mean difference, 0.11 [95% CI, 0.002-0.21] SDs) (Figure 3; eTable 5 in Supplement 1). No other associations were observed at the *P* < .05 level between AD trajectory and BMI, systolic blood pressure, total cholesterol, LDL-C, triglycerides, or cardiometabolic risk score.

Measures of subclinical atherosclerosis were similar among participants in each group (eTable 2 in Supplement 1). After adjustment, we found no consistent associations between AD phenotype and carotid intima media thickness or pulse wave velocity, except for a small increase in pulse wave velocity among participants in the moderate-declining group at age 17 years (mean difference, 0.11 [95% CI, 0.01-0.22] SDs) (Figure 3, eTable 5 in Supplement 1).

## Discussion

In this longitudinal population-based cohort study, we found no evidence that children with AD were at increased early cardiovascular risk. To assess the consistency of our results, we evaluated the association in multiple ways. For the exposure, we evaluated the period prevalence of AD, AD severity at each time point, and AD subtypes representing cumulative patterns of AD activity and severity from birth through adolescence. For the primary outcome, we examined the cardiometabolic risk score at ages 15 and 17 years. For secondary outcomes, we examined individual risk factors at multiple time points throughout childhood and adolescence and subclinical markers of atherosclerosis at ages 17 and 24 years. Across all of these analyses, we found no consistent evidence for elevated cardiovascular risk with any measure of AD, despite relatively narrow confidence intervals and lenient *P* values that were not adjusted for multiple testing. Narrow confidence intervals are important because they indicate that if other samples were collected, there is only a 5% chance that the true population mean difference would be outside this narrow range, potentially leading to different conclusions.^15^

Our results add to a limited number of studies in the literature on AD and CVD in childhood and early adulthood. A recent systematic review and meta-analysis^16^ found that among 10 publications, there was some evidence for an association with lipid disorders, but not across the entire population distribution (n = 7 studies; odds ratio, 1.24 [95% CI, 1.13-1.36]; [95% prediction interval, 0.95-1.61]). Only 4 of 10 studies included information on AD severity, and only 2 of 10 studies adjusted for important confounders, such as BMI, that we were able to address in this analysis. Moreover, in our analysis, we examined a more comprehensive cardiometabolic risk score and subclinical markers of atherosclerosis, in addition to traditional measures of cardiovascular risk, including blood pressure and lipid levels.

The American Heart Association has proposed more intensive screening recommendations and stringent therapeutic targets for children with chronic inflammatory diseases that have been identified as having increased risk for accelerated atherosclerosis and early CVD.^17^ The inflammatory conditions listed are relatively rare among US children and include juvenile inflammatory arthritis, systemic lupus erythematosus, inflammatory bowel disease, and HIV. AD, in contrast, affects up to 20% of children. Therefore, to the extent that our sample is generalizable, our results are important from a public health standpoint, suggesting that widespread screening of this population for early markers of cardiovascular risk is unlikely to identify individuals in need of early intervention.

The importance of inflammatory processes in the pathogenesis of CVD is now widely appreciated. Inflammatory markers can help predict individuals at highest risk of major cardiovascular outcomes, and new treatments focus on inflammatory targets, such as interleukin 6 (IL-6).^18,19^ Although AD has been associated with increased levels of inflammatory markers in other populations,^20,21^ members of our team previously found no association between IL-6 at age 9 years or C-reactive protein at ages 9, 16, or 18 years with AD (including severe AD) in a prior analysis of the ALSPAC cohort.^22^ The proportion of individuals with severe AD in the ALSPAC cohort is lower than among other population-based studies.^23^ Therefore, it is possible that additional research in other childhood populations with more participants with severe disease or elevated inflammatory markers will reveal an elevated risk in this subgroup.

Our results also highlight the importance of additional future research needed to understand the association between AD subtypes with CVD in adult populations. Data on the association between AD and CVD are mixed; the association is observed most often among those with more severe or active AD in adulthood.^1,5^ Although older reviews suggest that most AD begins in childhood, adult-onset disease is now recognized as common, and more research is needed to understand how age of AD onset or inflammatory phenotype impacts the association with CVD in adults.^24,25^

### Strengths and Limitations

The strengths of our study included a large, population-based birth cohort followed up for over 2 decades with repeated measures of AD activity and severity and multiple measures of cardiovascular risk. This study also has limitations, including attrition common to large long-term birth cohort studies and a lack of detailed skin measures. Although we cannot eliminate the possibility of bias, it is unlikely that dropout was highly correlated with AD, as the study was designed to assess childhood development more generally. We examined missing data patterns and adjusted for socioeconomic factors known to be associated with attrition from the cohort based on prior work.^26,27^ We did not correct for multiple testing, as we found no consistent evidence that AD was associated with cardiovascular risk in children. Additionally, prior work has validated the use of parental report of AD diagnosis and severity.^11^ Finally, the ALSPAC cohort is largely a White population; future work in more diverse populations will help to inform the generalizability of our results.

## Conclusions

In conclusion, this cohort study of children and adolescents in the UK with predominantly mild AD found no evidence for increased early cardiovascular risk in this population. These findings suggest that widespread screening of children and adolescents with AD for early markers of cardiovascular risk is unlikely to improve the identification of individuals in need of early intervention.

## Notes

Supplement 1. **eFigure 1.** Ages of cardiovascular risk and AD assessments **eFigure 2.** Prevalence of active AD at each time point by severity **eFigure 3.** Mean values of cardiovascular risk factors by age **eFigure 4.** Results of unadjusted cross-sectional analyses (standardized betas 95% CI) **eFigure 5.** Results of adjusted cross-sectional analyses (standardized betas 95% CI) **eFigure 6.** Results of the unadjusted longitudinal analyses showing the association between AD activity and severity subtype and CVD risk factors at ages 17 and 24 (standardized betas 95% CI) **eTable 1.** Distribution of participant characteristics between those included and excluded from analyses **eTable 2.** Average carotid intima media thickness (cIMT) and pulse wave velocity (PWV) by latent class group **eTable 3.** Results of cross-sectional analyses between AD and cardiovascular risk factors (standardized betas 95% CI) **eTable 4.** Results of cross-sectional analyses between AD severity and cardiovascular risk factors (standardized betas 95% CI) **eTable 5.** Results of longitudinal analyses showing the association between AD activity and severity subtype and CVD risk factors at ages 17 and 24 (standardized betas 95% CI) **eTable 6.** Sample size for models

Supplement 2.
Data Sharing Statement
