# Supplementary material for: Atopic Dermatitis and Markers of Early Cardiovascular Risk in Children and Adolescents
Source: JAMA Netw Open. 2026 Mar 24;9(3):e262962. doi: 10.1001/jamanetworkopen.2026.2962 (PMC13014201; doi:10.1001/jamanetworkopen.2026.2962)
Supplement: Supplement 1. — eFigure 1. Ages of cardiovascular risk and AD assessments eFigure 2. Prevalence of active AD at each time point by severity eFigure 3. Mean values of cardiovascular risk factors by age eFigure 4. Results of unadjusted cross-sectional analyses (standardized betas 95% CI) eFigure 5. Results of adjusted cross-sectional analyses (standardized betas 95% CI) eFigure 6. Results of the unadjusted longitudinal analyses showing the association between AD activity and severity subtype and CVD risk factors at ages 17 and 24 (standardized betas 95% CI) eTable 1. Distribution of participant characteristics between those included and excluded from analyses eTable 2. Average carotid intima media thickness (cIMT) and pulse wave velocity (PWV) by latent class group eTable 3. Results of cross-sectional analyses between AD and cardiovascular risk factors (standardized betas 95% CI) eTable 4. Results of cross-sectional analyses between AD severity and cardiovascular risk factors (standardized betas 95% CI) eTable 5. Results of longitudinal analyses showing the association between AD activity and severity subtype and CVD risk factors at ages 17 and 24 (standardized betas 95% CI) eTable 6. Sample size for models [file jamanetwopen-e262962-s001.pdf]

Supplementary Online Content

Ye M, McCulloch CE, Iribarren C, Langan SM, Abuabara K. Atopic dermatitis and markers of early cardiovascular risk in children and adolescents. *JAMA Netw Open*. 2026;9(3):e262962. doi:10.1001/jamanetworkopen.2026.2962

eFigure 1. Ages of cardiovascular risk and AD assessments
eFigure 2. Prevalence of active AD at each time point by severity
eFigure 3. Mean values of cardiovascular risk factors by age
eFigure 4. Results of unadjusted cross-sectional analyses (standardized betas 95% CI)
eFigure 5. Results of adjusted cross-sectional analyses (standardized betas 95% CI)
eFigure 6. Results of the unadjusted longitudinal analyses showing the association between AD activity and severity subtype and CVD risk factors at ages 17 and 24 (standardized betas 95% CI)
eTable 1. Distribution of participant characteristics between those included and excluded from analyses
eTable 2. Average carotid intima media thickness (cIMT) and pulse wave velocity (PWV) by latent class group
eTable 3. Results of cross-sectional analyses between AD and cardiovascular risk factors (standardized betas 95% CI)
eTable 4. Results of cross-sectional analyses between AD severity and cardiovascular risk factors (standardized betas 95% CI)
eTable 5. Results of longitudinal analyses showing the association between AD activity and severity subtype and CVD risk factors at ages 17 and 24 (standardized betas 95% CI)
eTable 6. Sample size for models

This supplementary material has been provided by the authors to give readers additional information about their work.

© 2026 Ye M et al. *JAMA Network Open*.

eFigure 1. Ages of cardiovascular risk and AD assessments

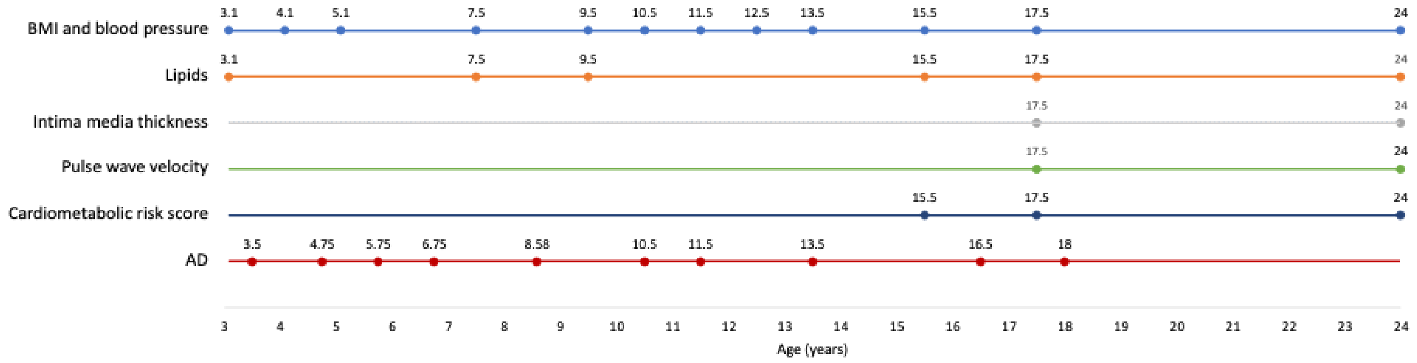

Abbreviations: AD-atopic dermatitis, BMI-body mass index

© 2026 Ye M et al. *JAMA Network Open*.

eFigure 2. Prevalence of active AD at each time point by severity

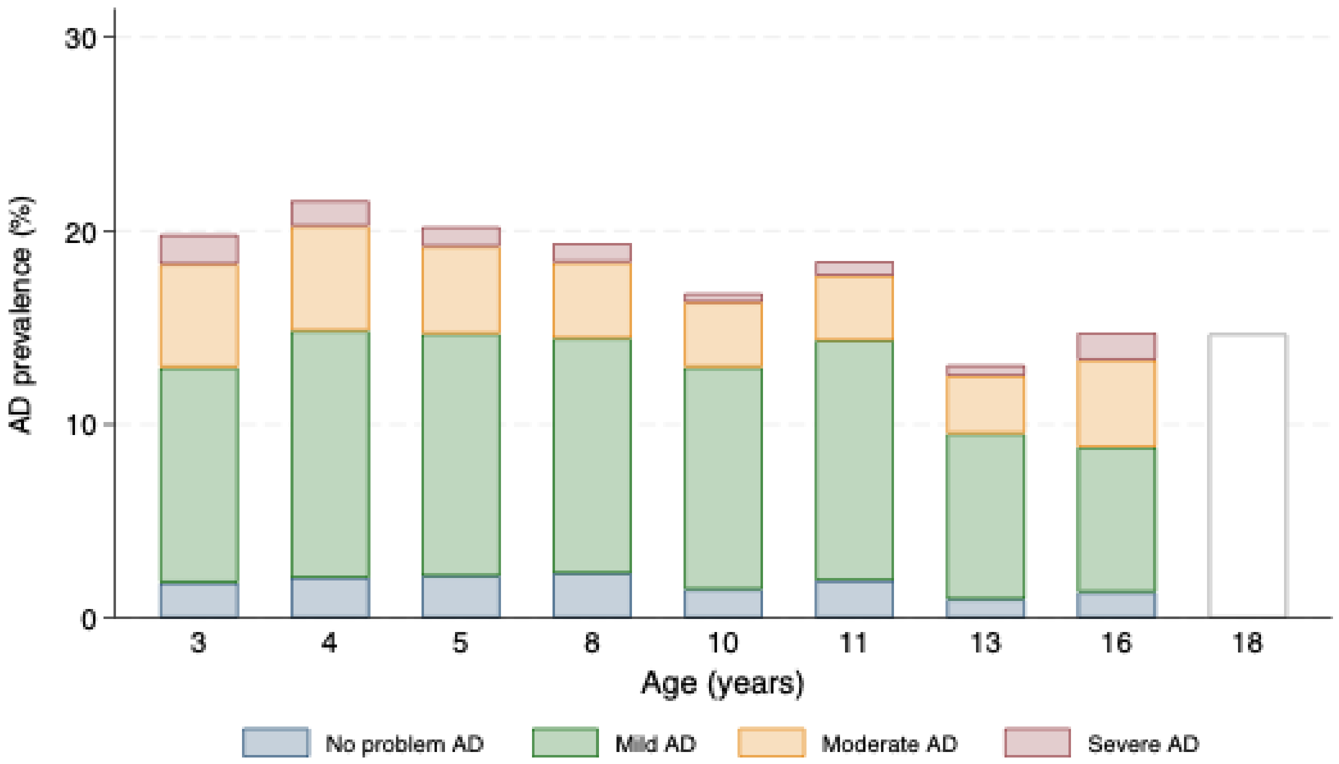

Note: At age 18, participants were not asked about AD severity
Abbreviations: AD-atopic dermatitis

© 2026 Ye M et al. *JAMA Network Open*.

eFigure 3. Mean values of cardiovascular risk factors by age

A. Blood pressure

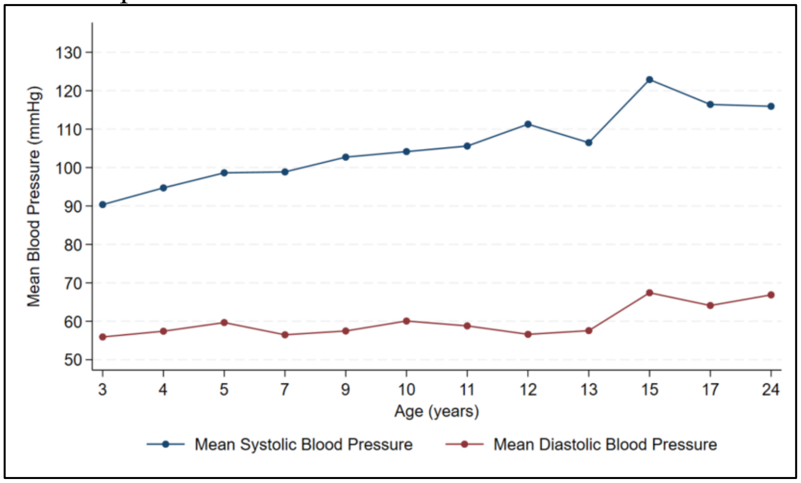

B. BMI

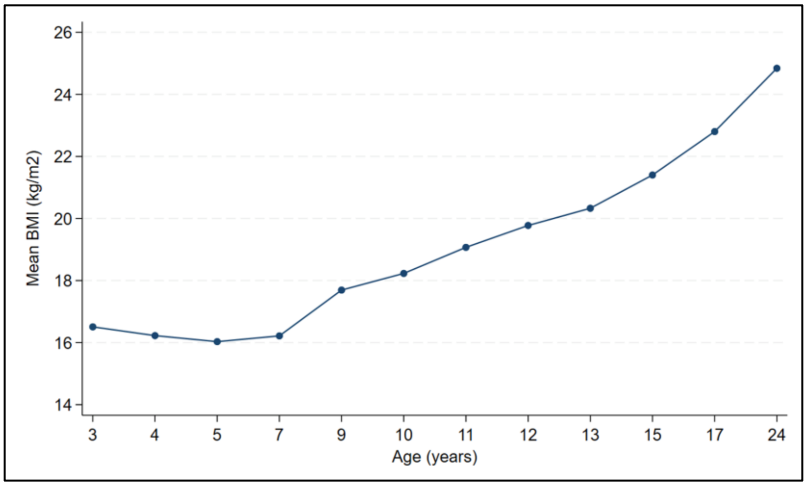

C. Lipids

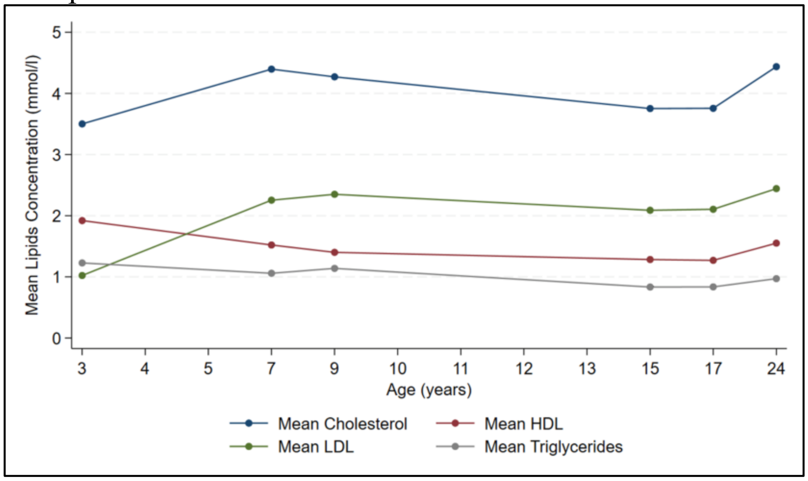

Abbreviations: BMI-body mass index, HDL- high-density lipoprotein, LDL- low-density lipoprotein, mmHg-millimeters of mercury, mmol/l-millimoles per liter

© 2026 Ye M et al. *JAMA Network Open*.

eFigure 4. Results of unadjusted cross-sectional analyses (standardized betas 95% CI)[a]

A. Cardiometabolic risk score

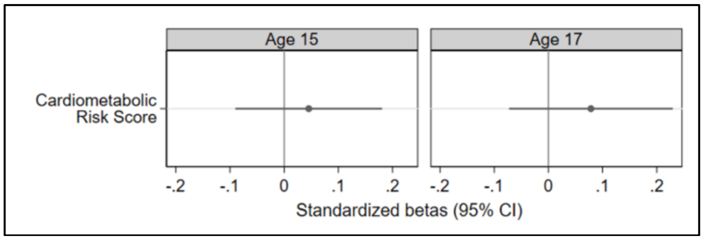

B. Blood pressure

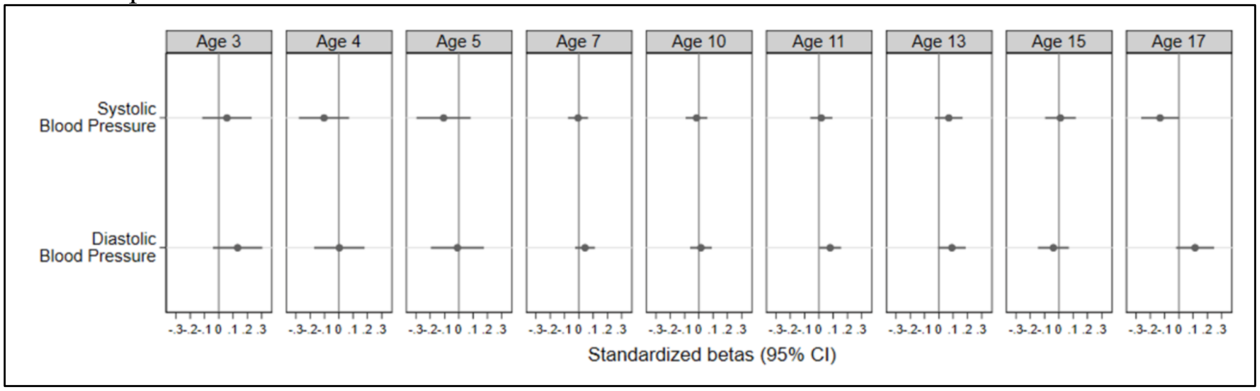

C. BMI

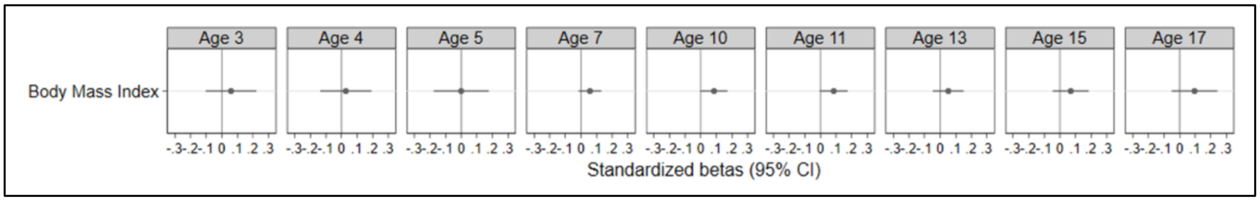

D. Lipids

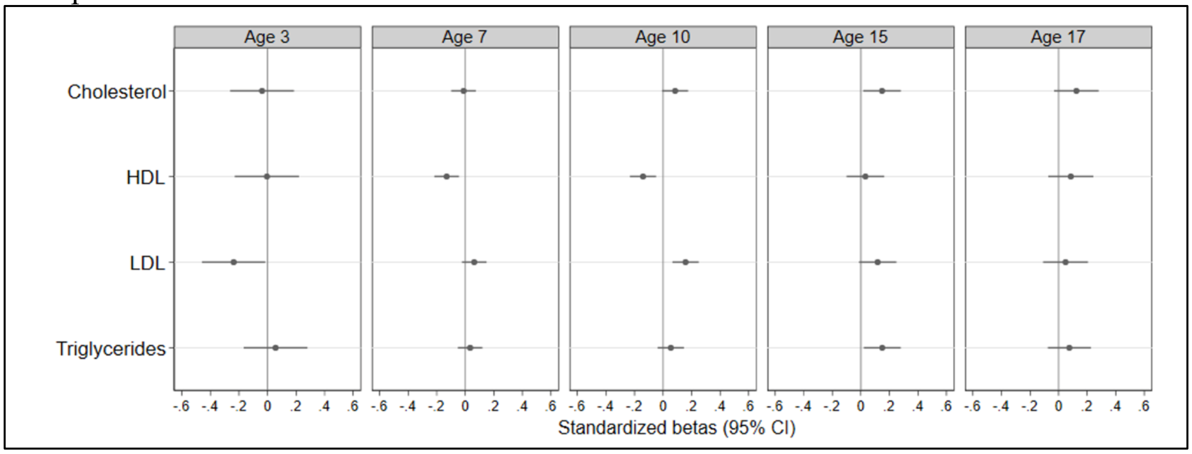

[a]Linear regressions showing the standardized betas in cardiovascular risk factors for atopic dermatitis as compared to no atopic dermatitis (reference group).
Abbreviations: BMI-body mass index, CI-confidence interval, HDL- high-density lipoprotein, LDL- low-density lipoprotein

© 2026 Ye M et al. *JAMA Network Open*.

eFigure 5. Results of adjusted cross-sectional analyses (standardized betas 95% CI)[a]

A. Cardiometabolic risk score

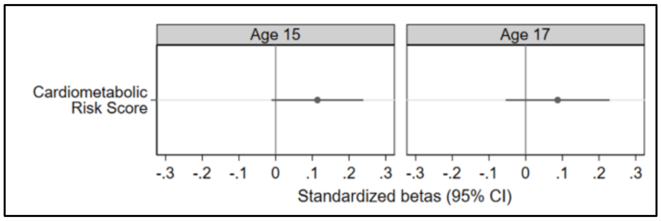

B. Blood pressure

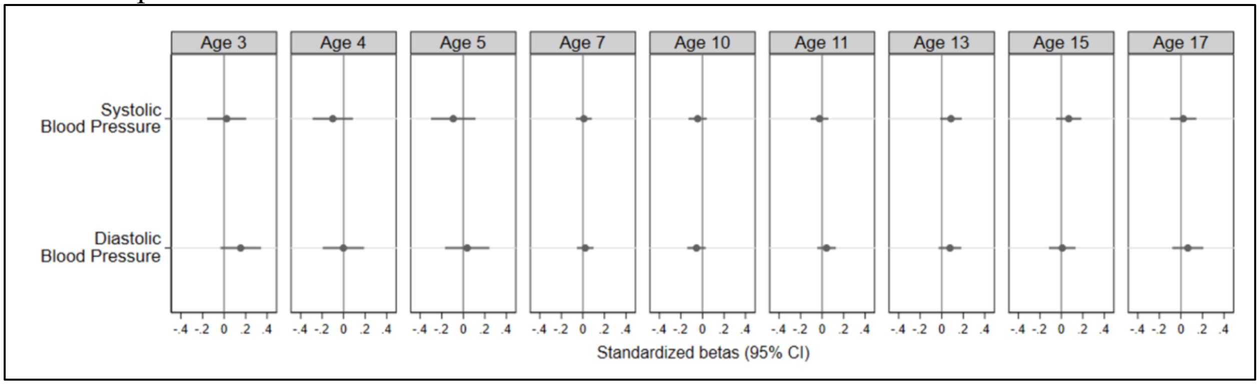

C. BMI

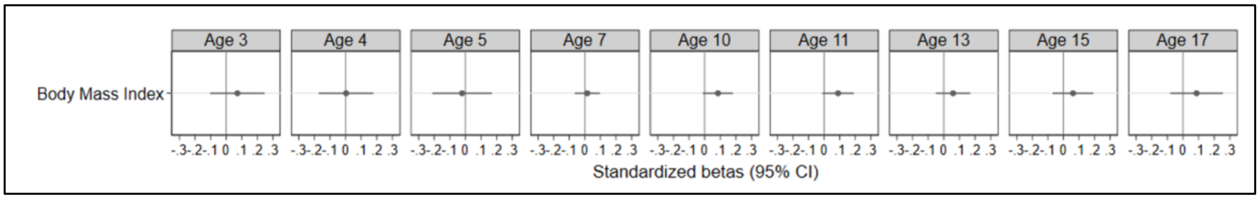

D. Lipids

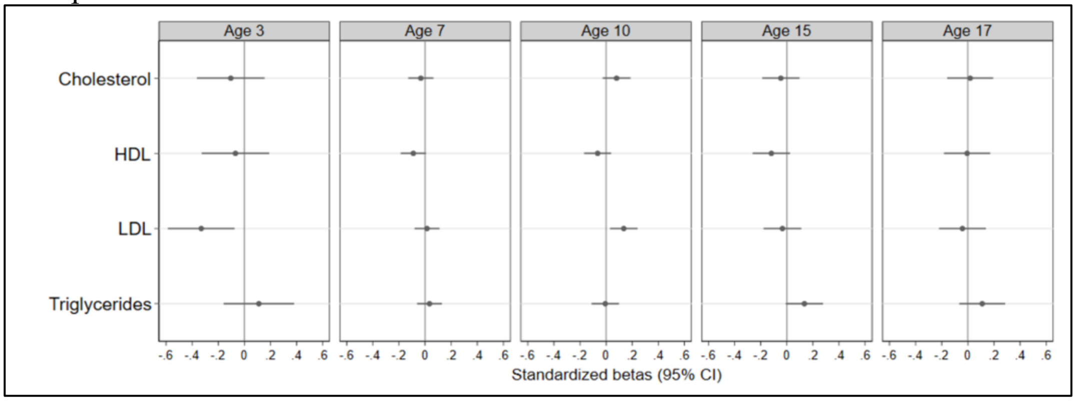

[a]Linear regressions showing the standardized betas in cardiovascular risk factors for atopic dermatitis as compared to no atopic dermatitis (reference group). All models were adjusted for sex, race/ethnicity, birthweight, maternal age at birth, gestational age, parity, socioeconomic status, maternal education, ever wheeze up to age of interest, ever hay fever up to age of interest, childhood tobacco smoke exposure, heaviness of traffic near home. All models, except for BMI, were also adjusted for BMI.

Abbreviations: BMI-body mass index, CI-confidence interval, HDL- high-density lipoprotein, LDL- low-density lipoprotein

© 2026 Ye M et al. *JAMA Network Open*.

eFigure 6. Results of the unadjusted longitudinal analyses showing the association between AD activity and severity subtype and CVD risk factors at ages 17 and 24 (standardized betas 95% CI)[a]

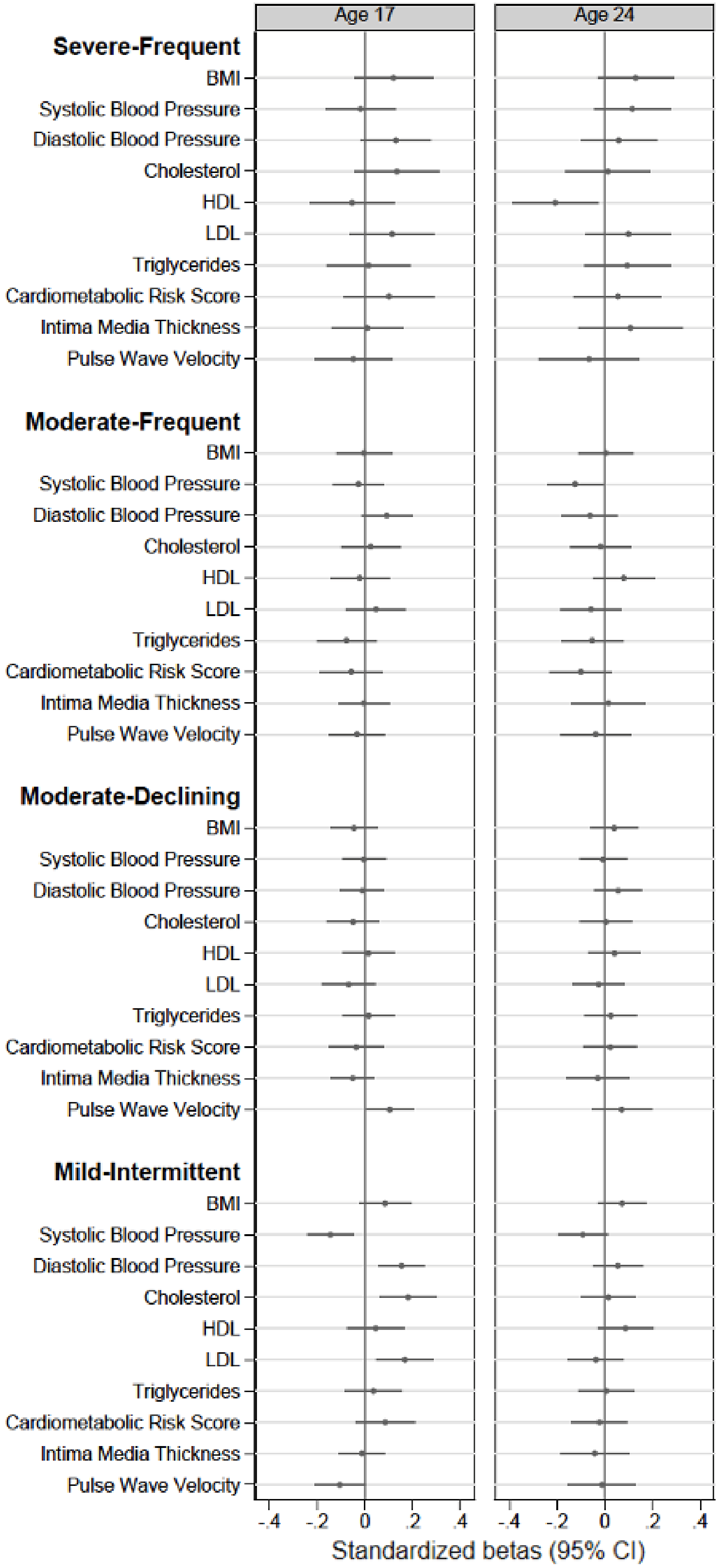

[a]Linear regressions showing the standardized betas in cardiovascular risk factors for Severe-Frequent, Moderate-Frequent, Moderate-Declining, and Mild-Intermittent atopic dermatitis activity and severity groups, as compared to the Unaffected/Rare atopic dermatitis group (reference group).

© 2026 Ye M et al. *JAMA Network Open*.

Abbreviations: BMI-body mass index, CI-confidence interval, HDL- high-density lipoprotein, LDL- low-density lipoprotein

© 2026 Ye M et al. *JAMA Network Open*.

eTable 1. Distribution of participant characteristics between those included and excluded from analyses

	Excluded from analyses* N=4666	Included in analyses* N=9281	Standardized mean difference
Sex			
Male	2513 (54.15)	4669 (50.31)	0.08
Female	2128 (45.85)	4612 (49.69)	
Race			
Non-white	236 (7.11)	372 (4.26)	0.12
White	3081 (92.89)	8366 (95.74)	
Low birthweight (<2500 grams)			
No	4270 (93.29)	8727 (95.22)	-0.08
Yes	307 (6.71)	438 (4.78)	
Maternal age at birth			
20 years or younger	626 (13.42)	378 (4.07)	0.33
21-34 years	3727 (79.88)	7835 (84.42)	-0.12
Older than 34 years	313 (6.71)	1068 (11.51)	-0.17
Pre-term birth (<37 weeks)			
No	4359 (93.42)	8745 (94.22)	-0.03
Yes	307 (6.58)	536 (5.78)	
Parity			
0	1685 (43.32)	4032 (45.66)	-0.05
1	1306 (33.57)	3140 (35.56)	-0.04
2+	899 (23.11)	1658 (18.78)	0.11
Parental Social class at 32 weeks gestation+3 years			
Professional	324 (10.12)	1539 (17.43)	-0.21
Managerial and technical	1315 (41.07)	4386 (49.68)	-0.17
Skilled non-manual	755 (23.58)	1832 (20.75)	0.07
Skilled manual	602 (18.80)	805 (9.12)	0.28
Partly skilled	172 (5.37)	223 (2.53)	0.15
Unskilled	34 (1.06)	43 (0.49)	0.07
Maternal education at 32 weeks gestation			
CSE/None	1154 (33.04)	1347 (15.14)	**0.42**
Vocational	399 (11.42)	825 (9.27)	0.07
O level	1129 (32.32)	3156 (35.48)	-0.07
A level	545 (15.60)	2237 (25.15)	-0.24
Degree (University or higher)	266 (7.62)	1330 (14.95)	-0.23
Passive smoke exposure per week at 6 months			
None	1670 (61.02)	6401 (74.73)	**-0.29**
Low (<1 hour)	348 (12.71)	922 (10.76)	0.06
Moderate (1-2 hours)	227 (8.29)	517 (6.04)	0.09
High (3+ hours)	492 (17.98)	725 (8.46)	0.28

© 2026 Ye M et al. *JAMA Network Open*.

Rating of traffic level at 8 months			
Very busy	251 (9.43)	634 (7.46)	0.07
Busy	350 (13.14)	1081 (12.72)	0.01
Moderate	805 (30.23)	2310 (27.17)	0.07
Quiet	774 (29.06)	2673 (31.44)	-0.05
Very quiet	483 (18.14)	1803 (21.21)	-0.08

*Values for each characteristic may not add up to the total because of missing data; percentages are of those with data. **Bold text** indicates nominal significance (i.e. p<0.05, not adjusted for multiple testing).
Abbreviations: CSE-Certificate of Secondary Education

© 2026 Ye M et al. *JAMA Network Open*.

eTable 2. Average carotid intima media thickness (cIMT) and pulse wave velocity (PWV) by latent class group

	Age 17		Age 24	
	cIMT, mean (SD)	PWV, mean (SD)	cIMT, mean (SD)	PWV, mean (SD)
Severe-Frequent	0.48 (0.04)	5.73 (0.62)	0.46 (0.05)	6.23 (0.95)
Moderate-Frequent	0.48 (0.04)	5.74 (0.65)	0.46 (0.05)	6.26 (1.01)
Moderate-Declining	0.47 (0.05)	5.84 (0.78)	0.46 (0.04)	6.38 (1.27)
Mild-Intermittent	0.48 (0.04)	5.69 (0.68)	0.46 (0.05)	6.29 (1.04)
Unaffected/Rare	0.48 (0.05)	5.76 (0.69)	0.46 (0.05)	6.31 (1.08)

Abbreviations: SD-standard deviation

© 2026 Ye M et al. *JAMA Network Open*.

eTable 3. Results of cross-sectional analyses between AD and cardiovascular risk factors (standardized betas 95% CI)[a]

	3 years	4 years	5 years	7 years	10 years	11 years	13 years	15 years	17 years
**Unadjusted**									
BMI	0.06 (-0.10, 0.22)	0.03 (-0.14, 0.19)	-0.001 (-0.18, 0.18)	0.06 (-0.02, 0.13)	0.09 (-0.0002, 0.17)	0.09 (-0.004, 0.17)	0.05 (-0.05, 0.15)	0.07 (-0.05, 0.19)	0.10 (-0.05, 0.24)
Systolic blood pressure	0.06 (-0.12, 0.23)	-0.10 (-0.28, 0.07)	-0.11 (-0.30, 0.08)	-0.01 (-0.08, 0.06)	-0.02 (-0.09, 0.06)	0.02 (-0.06, 0.09)	0.07 (-0.03, 0.17)	0.01 (-0.10, 0.12)	**-0.13 (-0.26, -0.00004)**
Diastolic blood pressure	0.13 (-0.04, 0.30)	0.003 (-0.17, 0.18)	-0.01 (-0.20, 0.18)	0.04 (-0.03, 0.11)	0.02 (-0.06, 0.09)	**0.08 (0.003, 0.16)**	0.09 (-0.005, 0.19)	-0.04 (-0.15, 0.07)	0.11 (-0.02, 0.25)
Total cholesterol (mmol/l)	-0.04 (-0.26, 0.19)	-	-	-0.01 (-0.10, 0.07)	0.08 (-0.01, 0.18)	-	-	**0.15 (0.02, 0.28)**	0.12 (-0.03, 0.28)
HDL (mmol/l)	-0.004 (-0.23, 0.22)	-	-	**-0.13 (-0.22, -0.04)**	**-0.14 (-0.23, -0.05)**	-	-	0.03 (-0.10, 0.16)	0.09 (-0.07, 0.24)
LDL (mmol/l)	**-0.24 (-0.46, -0.02)**	-	-	0.06 (-0.02, 0.15)	**0.16 (0.07, 0.25)**	-	-	0.12 (-0.01, 0.25)	0.05 (-0.11, 0.21)
Log Triglycerides (mmol/l)	0.06 (-0.17, 0.28)	-	-	0.03 (-0.05, 0.12)	0.05 (-0.04, 0.15)	-	-	**0.15 (0.02, 0.28)**	0.08 (-0.08, 0.23)
Cardiometabolic risk score	-	-	-	-	-	-	-	0.05 (-0.09, 0.18)	0.08 (-0.07, 0.23)
**Adjusted[b]**									
BMI	0.07 (-0.10, 0.25)	0.003 (-0.17, 0.18)	-0.02 (-0.21, 0.17)	0.02 (-0.07, 0.10)	0.09 (-0.01, 0.18)	0.09 (-0.01, 0.19)	0.06 (-0.05, 0.17)	0.06 (-0.07, 0.19)	0.09 (-0.08, 0.26)
Systolic blood pressure	0.02 (-0.16, 0.21)	-0.10 (-0.29, 0.09)	-0.09 (-0.30, 0.11)	0.01 (-0.06, 0.08)	-0.04 (-0.13, 0.04)	-0.02 (-0.11, 0.06)	0.09 (-0.01, 0.19)	0.07 (-0.05, 0.19)	0.02 (-0.10, 0.14)
Diastolic blood pressure	0.15 (-0.03, 0.34)	-0.002 (-0.19, 0.19)	0.04 (-0.17, 0.24)	0.02 (-0.05, 0.10)	-0.06 (-0.14, 0.03)	0.04 (-0.04, 0.13)	0.08 (-0.03, 0.18)	0.01 (-0.11, 0.13)	0.06 (-0.08, 0.21)
Total cholesterol (mmol/l)	-0.10 (-0.36, 0.16)	-	-	-0.03 (-0.13, 0.06)	0.08 (-0.03, 0.19)	-	-	-0.05 (-0.19, 0.10)	0.02 (-0.16, 0.19)
HDL (mmol/l)	-0.07 (-0.33, 0.19)	-	-	-0.09 (-0.19, 0.01)	-0.06 (-0.17, 0.04)	-	-	-0.12 (-0.26, 0.02)	-0.01 (-0.18, 0.17)
LDL (mmol/l)	**-0.33 (-0.58, -0.07)**	-	-	0.01 (-0.08, 0.11)	**0.14 (0.03, 0.24)**	-	-	-0.03 (-0.18, 0.11)	-0.04 (-0.22, 0.14)
Log Triglycerides (mmol/l)	0.11 (-0.16, 0.38)	-	-	0.03 (-0.06, 0.13)	-0.01 (-0.11, 0.10)	-	-	0.13 (-0.01, 0.28)	0.11 (-0.07, 0.29)
Cardiometabolic risk score	-	-	-	-	-	-	-	0.11 (-0.01, 0.24)	0.09 (-0.05, 0.23)

Notes: Bold text indicates nominal significance (i.e. p<0.05, not adjusted for multiple testing). [a]Linear regressions showing the standardized betas in cardiovascular risk factors for atopic dermatitis as compared to no atopic dermatitis (reference group). [b]Adjusted for sex, race/ethnicity, birthweight, maternal age at birth, gestational age, parity, socioeconomic status, maternal education, ever wheeze up to age of interest, ever hay fever up to age of interest, childhood tobacco smoke exposure, heaviness of traffic near home. All models, except for BMI, were also adjusted for BMI.
Abbreviations: BMI-body mass index, CI-confidence interval, HDL- high-density lipoprotein, LDL- low-density lipoprotein, mmol/l-millimoles per liter

© 2026 Ye M et al. *JAMA Network Open*.

eTable 4. Results of cross-sectional analyses between AD severity and cardiovascular risk factors (standardized betas 95% CI)[a]

	3 years	4 years	5 years	7 years	10 years	11 years	13 years	15 years
BMI								
No problem/mild AD	0.03 (-0.17, 0.23)	-0.02 (-0.22, 0.18)	0.07 (-0.14, 0.28)	-0.03 (-0.12, 0.06)	0.06 (-0.04, 0.17)	0.04 (-0.06, 0.15)	0.06 (-0.06, 0.18)	0.09 (-0.07, 0.25)
Moderate/severe AD	0.16 (-0.10, 0.42)	0.05 (-0.21, 0.31)	-0.27 (-0.58, 0.03)	**0.15 (0.01, 0.29)**	0.17 (-0.002, 0.35)	**0.26 (0.07, 0.44)**	0.06 (-0.13, 0.25)	0.03 (-0.16, 0.21)
Systolic blood pressure								
No problem/mild AD	-0.03 (-0.23, 0.18)	-0.13 (-0.34, 0.08)	-0.07 (-0.30, 0.16)	-0.002 (-0.08, 0.08)	-0.05 (-0.14, 0.04)	-0.04 (-0.13, 0.05)	0.08 (-0.04, 0.19)	0.08 (-0.06, 0.22)
Moderate/severe AD	0.12 (-0.15, 0.40)	-0.04 (-0.31, 0.23)	-0.14 (-0.47, 0.18)	0.04 (-0.08, 0.17)	-0.03 (-0.18, 0.12)	0.03 (-0.12, 0.18)	0.11 (-0.06, 0.28)	0.08 (-0.09, 0.24)
Diastolic blood pressure								
No problem/mild AD	0.18 (-0.03, 0.40)	-0.10 (-0.31, 0.12)	0.02 (-0.21, 0.25)	0.02 (-0.07, 0.10)	-0.08 (-0.18, 0.01)	0.01 (-0.09, 0.10)	0.03 (-0.09, 0.15)	0.08 (-0.07, 0.23)
Moderate/severe AD	0.09 (-0.19, 0.38)	0.17 (-0.10, 0.45)	0.08 (-0.24, 0.40)	0.04 (-0.09, 0.17)	0.03 (-0.12, 0.19)	**0.17 (0.01, 0.32)**	**0.19 (0.01, 0.37)**	-0.09 (-0.26, 0.08)
Total cholesterol (mmol/l)								
No problem/mild AD	-0.01 (-0.31, 0.29)	-	-	-0.03 (-0.14, 0.07)	0.07 (-0.05, 0.18)	-	-	**-0.20 (-0.37, -0.03)**
Moderate/severe AD	-0.27 (-0.65, 0.10)	-	-	-0.04 (-0.21, 0.13)	0.14 (-0.06, 0.34)	-	-	0.17 (-0.02, 0.37)
HDL (mmol/l)								
No problem/mild AD	-0.04 (-0.34, 0.27)	-	-	-0.08 (-0.18, 0.03)	-0.06 (-0.17, 0.05)	-	-	-0.11 (-0.29, 0.06)
Moderate/severe AD	-0.12 (-0.50, 0.25)	-	-	-0.13 (-0.30, 0.04)	-0.08 (-0.27, 0.11)	-	-	-0.13 (-0.32, 0.07)
LDL (mmol/l)								
No problem/mild AD	**-0.32 (-0.61, -0.02)**	-	-	-0.01 (-0.11, 0.10)	0.11 (-0.002, 0.23)	-	-	**-0.20 (-0.37, -0.03)**
Moderate/severe AD	-0.35 (-0.72, 0.02)	-	-	0.08 (-0.09, 0.24)	**0.23 (0.03, 0.42)**	-	-	**0.20 (0.001, 0.40)**
Log Triglycerides (mmol/l)								
No problem/mild AD	0.27 (-0.04, 0.59)	-	-	0.05 (-0.05, 0.15)	-0.003 (-0.12, 0.11)	-	-	0.06 (-0.11, 0.23)
Moderate/severe AD	-0.18 (-0.57, 0.21)	-	-	-0.03 (-0.20, 0.13)	-0.005 (-0.20, 0.19)	-	-	**0.24 (0.05, 0.44)**
Cardiometabolic risk score								
No problem/mild AD	-	-	-	-	-	-	-	0.12 (-0.03, 0.27)
Moderate/severe AD	-	-	-	-	-	-	-	0.11 (-0.06, 0.28)

Notes: Bold text indicates nominal significance (i.e. p<0.05, not adjusted for multiple testing). [a]Linear regressions showing the standardized betas in cardiovascular risk factors for no problem/mild and moderate/severe atopic dermatitis as compared to no atopic dermatitis (reference group). All models were adjusted for sex, race/ethnicity, birthweight, maternal age at birth, gestational age, parity, socioeconomic status, maternal education, ever wheeze up to age of interest, ever hay fever up to age of interest, childhood tobacco smoke exposure, heaviness of traffic near home. All models, except for BMI, were also adjusted for BMI.

© 2026 Ye M et al. *JAMA Network Open.*

Abbreviations: BMI-body mass index, CI-confidence interval, HDL- high-density lipoprotein, LDL- low-density lipoprotein, mmol/l-millimoles per liter

© 2026 Ye M et al. *JAMA Network Open*.

eTable 5. Results of longitudinal analyses showing the association between AD activity and severity subtype and CVD risk factors at ages 17 and 24 (standardized betas 95% CI)[a]

	Age 17				Age 24			
	Severe-Frequent	Moderate-Frequent	Moderate-Declining	Mild-Intermittent	Severe-Frequent	Moderate-Frequent	Moderate-Declining	Mild-Intermittent
**Unadjusted**								
BMI	0.12 (-0.04, 0.28)	-0.004 (-0.12, 0.11)	-0.05 (-0.15, 0.05)	0.08 (-0.02, 0.19)	0.13 (-0.03, 0.29)	0.005 (-0.11, 0.12)	0.04 (-0.06, 0.14)	0.07 (-0.03, 0.18)
Systolic blood pressure	-0.02 (-0.17, 0.13)	-0.03 (-0.13, 0.08)	-0.004 (-0.09, 0.09)	**-0.14 (-0.24, -0.05)**	0.12 (-0.04, 0.28)	**-0.12 (-0.24, -0.01)**	-0.01 (-0.11, 0.09)	-0.09 (-0.19, 0.01)
Diastolic blood pressure	0.13 (-0.02, 0.28)	0.09 (-0.01, 0.20)	-0.01 (-0.10, 0.08)	**0.15 (0.06, 0.25)**	0.06 (-0.10, 0.22)	-0.06 (-0.18, 0.05)	0.06 (-0.04, 0.15)	0.06 (-0.05, 0.16)
Total cholesterol (mmol/l)	0.13 (-0.04, 0.31)	0.02 (-0.10, 0.15)	-0.05 (-0.16, 0.06)	**0.18 (0.06, 0.30)**	0.01 (-0.16, 0.19)	-0.02 (-0.15, 0.11)	0.005 (-0.10, 0.11)	0.02 (-0.10, 0.13)
HDL (mmol/l)	-0.05 (-0.23, 0.12)	-0.02 (-0.15, 0.10)	0.01 (-0.10, 0.13)	0.05 (-0.07, 0.17)	**-0.21 (-0.39, -0.03)**	0.08 (-0.05, 0.21)	0.04 (-0.07, 0.15)	0.09 (-0.03, 0.20)
LDL (mmol/l)	0.11 (-0.06, 0.29)	0.05 (-0.08, 0.17)	-0.07 (-0.18, 0.04)	**0.17 (0.05, 0.29)**	0.10 (-0.08, 0.28)	-0.06 (-0.19, 0.07)	-0.02 (-0.13, 0.08)	-0.04 (-0.15 0.08)
Log Triglycerides (mmol/l)	0.02 (-0.16, 0.19)	-0.08 (-0.20, 0.05)	0.02 (-0.09, 0.13)	0.04 (-0.08, 0.16)	0.09 (-0.08, 0.27)	-0.05 (-0.18, 0.08)	0.03 (-0.08, 0.13)	0.01 (-0.11, 0.12)
Intima media thickness	0.01 (-0.14, 0.16)	-0.004 (-0.11, 0.10)	-0.05 (-0.14, 0.04)	-0.01 (-0.11, 0.08)	0.11 (-0.11, 0.33)	0.02 (-0.14, 0.17)	-0.03 (-0.16, 0.10)	-0.04 (-0.19, 0.10)
Pulse wave velocity	-0.05 (-0.21, 0.11)	-0.03 (-0.15, 0.08)	**0.10 (0.01, 0.20)**	-0.11 (-0.21, 0.001)	-0.07 (-0.28, 0.14)	-0.04 (-0.18, 0.11)	0.07 (-0.06, 0.20)	-0.01 (-0.15, 0.13)
Cardiometabolic risk score	0.10 (-0.09, 0.29)	-0.06 (-0.19, 0.07)	-0.04 (-0.15, 0.08)	0.09 (-0.04, 0.21)	0.06 (-0.13, 0.24)	-0.10 (-0.23, 0.03)	0.02 (-0.09, 0.13)	-0.02 (-0.14, 0.09)
**Adjusted[b]**								
BMI	0.05 (-0.15, 0.24)	-0.004 (-0.13, 0.13)	-0.02 (-0.13, 0.10)	0.11 (-0.01, 0.24)	0.07 (-0.11, 0.25)	0.02 (-0.11, 0.15)	0.07 (-0.04, 0.18)	0.10 (-0.01, 0.21)
Systolic blood pressure	-0.05 (-0.18, 0.09)	0.04 (-0.06, 0.13)	-0.03 (-0.12, 0.05)	0.03 (-0.05, 0.12)	0.09 (-0.06, 0.24)	-0.03 (-0.14, 0.07)	-0.06 (-0.15, 0.03)	-0.003 (-0.10, 0.09)
Diastolic blood pressure	0.02 (-0.15, 0.18)	0.05 (-0.07, 0.16)	0.03 (-0.07, 0.13)	**0.11 (0.002, 0.21)**	0.04 (-0.13, 0.21)	-0.04 (-0.16, 0.08)	0.05 (-0.06, 0.15)	0.03 (-0.08, 0.14)
Total cholesterol (mmol/l)	-0.02 (-0.22, 0.19)	-0.03 (-0.16, 0.10)	-0.02 (-0.14, 0.10)	0.07 (-0.06, 0.21)	-0.06 (-0.26, 0.14)	-0.06 (-0.20, 0.09)	0.02 (-0.11, 0.14)	-0.05 (-0.18, 0.08)
HDL (mmol/l)	-0.09 (-0.29, 0.12)	**-0.15 (-0.28, -0.02)**	-0.003 (-0.12, 0.12)	-0.04 (-0.17, 0.10)	**-0.24 (-0.42, -0.05)**	-0.05 (-0.18, 0.08)	0.08 (-0.03, 0.20)	0.03 (-0.09, 0.15)
LDL (mmol/l)	-0.01 (-0.22, 0.20)	0.04 (-0.09, 0.18)	-0.03 (-0.15, 0.10)	0.10 (-0.04, 0.23)	0.05 (-0.15, 0.24)	-0.03 (-0.17, 0.11)	-0.03 (-0.15, 0.09)	-0.05 (-0.18, 0.07)
Log Triglycerides (mmol/l)	-0.09 (-0.30, 0.12)	-0.04 (-0.18, 0.10)	0.01 (-0.11, 0.14)	-0.01 (-0.15, 0.12)	0.04 (-0.15, 0.23)	-0.03 (-0.17, 0.11)	0.01 (-0.11, 0.13)	-0.05 (-0.18, 0.07)
Intima media thickness	-0.003 (-0.17, 0.17)	0.02 (-0.10, 0.14)	0.02 (-0.08, 0.12)	0.07 (-0.04, 0.18)	0.01 (-0.24, 0.25)	0.06 (-0.11, 0.23)	-0.08 (-0.23, 0.07)	0.01 (-0.15, 0.17)
Pulse wave velocity	0.01 (-0.16, 0.19)	0.07 (-0.06, 0.19)	**0.11 (0.01, 0.22)**	0.07 (-0.04, 0.19)	-0.01 (-0.25, 0.23)	0.05 (-0.11, 0.21)	0.09 (-0.05, 0.23)	0.09 (-0.07, 0.25)
Cardiometabolic risk score	0.03 (-0.15, 0.21)	-0.02 (-0.14, 0.10)	-0.003 (-0.11, 0.10)	0.07 (-0.05, 0.19)	0.04 (-0.10, 0.18)	-0.01 (-0.11, 0.09)	-0.04 (-0.12, 0.05)	-0.02 (-0.11, 0.07)

© 2026 Ye M et al. *JAMA Network Open.*

Notes: Bold text indicates nominal significance (i.e. p<0.05, not adjusted for multiple testing). [a] Linear regressions showing the standardized betas in cardiovascular risk factors for Severe-Frequent, Moderate-Frequent, Moderate-Declining, and Mild-Intermittent atopic dermatitis activity and severity groups, as compared to the Unaffected/Rare atopic dermatitis group (reference group). [b]Adjusted for sex, race/ethnicity, birthweight, maternal age at birth, gestational age, parity, socioeconomic status, maternal education, ever wheeze up to age of interest, ever hay fever up to age of interest, childhood tobacco smoke exposure, heaviness of traffic near home. All models, except for BMI, were also adjusted for BMI.
Abbreviations: BMI-body mass index, CI-confidence interval, HDL- high-density lipoprotein, LDL- low-density lipoprotein, mmol/l-millimoles per liter

© 2026 Ye M et al. *JAMA Network Open*.

eTable 6. Sample size for models

**Cross-sectional unadjusted models**	3 years	4 years	5 years	7 years	10 years	11 years	13 years	15 years	17 years	24 years
BMI	955	903	836	6,306	5,959	5,596	4,958	3,459	2,340	-
Systolic blood pressure	926	851	787	6,199	5,745	5,543	4,339	3,393	2,215	-
Diastolic blood pressure	926	851	788	6,198	5,745	5,543	4,339	3,393	2,215	-
Total cholesterol (mmol/l)	570	-	-	4,192	4,020	-	-	2,275	1,563	-
HDL (mmol/l)	566	-	-	4,192	4,020	-	-	2,275	1,563	-
LDL (mmol/l)	570	-	-	4,192	4,019	-	-	2,275	1,563	-
Triglycerides (mmol/l)	570	-	-	4,192	4,020	-	-	2,275	1,563	-
Cardiometabolic risk score	-	-	-	-	-	-	-	2,126	1,439	-
**Cross-sectional adjusted models**										
BMI	828	768	709	5,210	4,729	4,440	3,936	2,787	1,893	-
Systolic blood pressure	798	718	667	5,087	4,505	4,371	3,438	2,707	1,779	-
Diastolic blood pressure	798	718	667	5,086	4,505	4,371	3,438	2,707	1,779	-
Total cholesterol (mmol/l)	460	-	-	3,446	3,057	-	-	1,820	1,233	-
HDL (mmol/l)	457	-	-	3,446	3,057	-	-	1,820	1,233	-
LDL (mmol/l)	460	-	-	3,446	3,056	-	-	1,820	1,233	-
Triglycerides (mmol/l)	460	-	-	3,446	3,057	-	-	1,820	1,233	-
Cardiometabolic risk score	-	-	-	-	-	-	-	1,733	1,161	-
**Longitudinal unadjusted models**	3 years	4 years	5 years	7-8 years	9-10 years	11 years	13 years	15-16 years	17-18 years	24 years
BMI	-	-	-	-	-	-	-	-	4,612	3,586
Systolic blood pressure	-	-	-	-	-	-	-	-	4,282	3,606
Diastolic blood pressure	-	-	-	-	-	-	-	-	4,282	3,606
Total cholesterol (mmol/l)	-	-	-	-	-	-	-	-	3,017	2,943
HDL (mmol/l)	-	-	-	-	-	-	-	-	3,017	2,943
LDL (mmol/l)	-	-	-	-	-	-	-	-	3,017	2,942

© 2026 Ye M et al. *JAMA Network Open*.

Triglycerides (mmol/l)	-	-	-	-	-	-	-	-	3,017	2,943
Intima media thickness	-	-	-	-	-	-	-	-	4,280	1,938
Pulse wave velocity	-	-	-	-	-	-	-	-	3,592	2,117
Cardiometabolic risk score	-	-	-	-	-	-	-	-	2,727	2,836
**Longitudinal adjusted models**										
BMI	-	-	-	-	-	-	-	-	3,379	2,666
Systolic blood pressure	-	-	-	-	-	-	-	-	3,118	2,657
Diastolic blood pressure	-	-	-	-	-	-	-	-	3,118	2,657
Total cholesterol (mmol/l)	-	-	-	-	-	-	-	-	2,191	2,179
HDL (mmol/l)	-	-	-	-	-	-	-	-	2,191	2,179
LDL (mmol/l)	-	-	-	-	-	-	-	-	2,191	2,178
Triglycerides (mmol/l)	-	-	-	-	-	-	-	-	2,191	2,178
Intima media thickness	-	-	-	-	-	-	-	-	3,113	1,494
Pulse wave velocity	-	-	-	-	-	-	-	-	2,682	1,628
Cardiometabolic risk score	-	-	-	-	-	-	-	-	2,039	2,128

Abbreviations: BMI-body mass index, HDL- high-density lipoprotein, LDL- low-density lipoprotein, mmol/l-millimoles per liter

© 2026 Ye M et al. *JAMA Network Open*.
