# Supplementary material for: Atopic Dermatitis and Markers of Early Cardiovascular Risk in Children and Adolescents
Source: JAMA Netw Open. 2026 Mar 24;9(3):e262962. doi: 10.1001/jamanetworkopen.2026.2962 (PMC13014201; doi:10.1001/jamanetworkopen.2026.2962)
Supplement: Supplement 2. — Data Sharing Statement [file jamanetwopen-e262962-s002.pdf]

Data Sharing Statement

Ye. Atopic Dermatitis and Markers of Early Cardiovascular Risk in Children and Adolescents. *JAMA Netw Open*. Published March 24, 2026. doi:10.1001/jamanetworkopen.2026.2962

Data
Data available: No

Additional Information
Explanation for why data not available: The data that support the findings of this study are available from ALSPAC. Restrictions apply to the availability of these data, which were used under license for this study. Data from ALSPAC is available following application to ALSPAC (https://www.bristol.ac.uk/alspac/researchers/access/).
